# Microbial interventions in yak colibacillosis: Lactobacillus-mediated regulation of intestinal barrier

**DOI:** 10.3389/fcimb.2024.1337439

**Published:** 2024-02-06

**Authors:** Jingbo Zhang, Xiaoli Ren, Shuo Wang, Ruidong Liu, Bin Shi, Hailong Dong, Qingxia Wu

**Affiliations:** ^1^ College of Animal Science, Tibet Agriculture and Animal Husbandry University, Linzhi, China; ^2^ Institute of Animal Husbandry and Veterinary Medicine, Tibet Autonomous Region Academy of Agriculture and Animal Science, Lhasa, China

**Keywords:** yak, Lactobacillus, Zonulin, intestinal barrier, tight junction

## Abstract

**Introduction:**

The etiology of Escherichia coli in yaks, along with its drug resistance, results in economic losses within the yak breeding industry. The utilization of lactic acid bacteria treatment has emerged as a viable alternative to antibiotics in managing colibacillosis.

**Methods:**

To elucidate the therapeutic mechanisms of Lactobacillus against Escherichia coli-induced intestinal barrier damage in yaks, we employed yak epithelial cells as the experimental model and established a monolayer epithelial barrier using Transwell. The study encompassed four groups: a control group, a model group (exposed to E. coli O78), a low-dose Lactobacillus group (*E. coli* O78 + 1 × 10^5^CFU LAB), and a high-dose Lactobacillus group (*E. coli* O78 + 1 × 10^7^CFU LAB). Various techniques, including transmembrane resistance measurement, CFU counting, RT-qPCR, and Western Blot, were employed to assess indicators related to cell barrier permeability and tight junction integrity.

**Results:**

In the Model group, Escherichia coli O78 significantly compromised the permeability and tight junction integrity of the yak epithelial barrier. It resulted in decreased transmembrane resistance, elevated FD4 flux, and bacterial translocation. Furthermore, it downregulated the mRNA and protein expression of MUC2, Occludin, and ZO-1, while upregulating the mRNA expression and protein expression of FABP2 and Zonulin, thereby impairing intestinal barrier function. Contrastingly, Lactobacillus exhibited a remarkable protective effect. It substantially increased transmembrane resistance, mitigated FD4 flux, and reduced bacterial translocation. Moreover, it significantly upregulated the mRNA and protein expression of MUC2, Occludin, and ZO-1, while downregulating the mRNA and protein expression of FABP2 and Zonulin. Notably, high-dose LAB demonstrated superior regulatory effects compared to the low-dose LAB group.

**Discussion:**

In conclusion, our findings suggest that Lactobacillus holds promise in treating yak colibacillosis by enhancing mucin and tight junction protein expression. Furthermore, we propose that Lactobacillus achieves these effects through the regulation of Zonulin.

## 1 Introduction

Yak, a cattle species indigenous to the Qinghai-Tibet Plateau, holds a predominant position in Xizang’s animal husbandry, constituting a substantial portion of the region’s livestock (Liu et al., 2023). Yak colibacillosis, an infectious disease triggered by Escherichia coli (*E. coli*) infection, manifests with typical symptoms such as high fever and diarrhea. It exhibits a high incidence and mortality rate, particularly affecting calf yaks and female yaks. The associated diarrhea poses a significant challenge in the yak breeding industry in recent years. (Rehman et al., 2017). The gastrointestinal tract of adult ruminants undergoes colonization by diverse microorganisms, comprising bacteria, archaea, viruses, protozoa and fungi, culminating in the formation of intestinal microbial barriers (Furman et al., 2020). The colonization of microorganisms in young individuals exerts a lasting influence on the rumen microbial population (Guo et al., 2020). In the case of female yaks, the intestinal microflora they carry is transmitted to newborn calves, influencing the establishment of the calves’ intestinal microbiota. Genomic analysis of fecal samples from pre-weaning calves and lactating cows reveals distinctions in the fecal bacterial community, underscoring the impact of age on microbial composition (Haley et al., 2020), Utilizing 16SrRNAsequencing to investigate the gastrointestinal microflora of goats, it was observed that young goats exhibited heightened sensitivity to changes in gastrointestinal flora, rendering them more susceptible to diarrhea symptoms (Wang et al., 2018). This indicates that environmental bacteria acquired by calves have the potential to disrupt intestinal microflora, leading to imbalances and subsequent diarrhea. Gastrointestinal microflora play a crucial role in protecting hosts from external threats and diseases through various mechanisms while also providing nitrogen sources for ruminants. Disturbances in this symbiotic relationship can result in gastrointestinal diseases such as acidosis, nutritional poisoning, abdominal distension and diarrhea (Belanche et al., 2021). Presently, the conventional approach to treating yak colibacillosis involves antibiotic therapy, which, over an extended period, has given rise to concerns related to drug residues and the disruption of intestinal flora (Jia et al., 2018; Dong et al., 2020). Given the intricate physiological structure of the gastrointestinal system in ruminants, solely relying on antibiotic therapy proves insufficient in addressing gastrointestinal flora-related ailments. The escalation in the detection of drug-resistant strains of *E. coli* in recent years has further compounded the challenges associated with preventing and treating yak colibacillosis.

The intestinal barrier serves as a defensive structure that demarcates sterile tissue *in vivo* from the microbiota *in vitro*. Its core mechanism lies in selective permeability (Felipe-López et al., 2023; Rogers et al., 2023). The transport of intestinal molecules from the intestinal lumen to the lamina propria involves two types of mechanisms: membrane receptor-mediated transcellular transport, including Transcellular transport, and Taracellular transport through the intercellular space regulated by Tight Junction (TJ) (Mowat et al., 2004). The Tight Junction of intestinal epithelial cells stands as the primary structure that constitutes the epithelial barrier. Its principal function is to maintain the surface polarity of intestinal epithelial cells, reversibly preventing the diffusion of macromolecules and microorganisms both inside and outside the epithelium (Kucharzik et al., 2001; Yu and Yang, 2009). Numerous proteins contribute to the integrity of the intestinal barrier and tight junction structure. Mucins MUC1 and MUC2 can adhere to pathogenic bacteria, safeguarding the intestinal epithelial mucosa (Cox et al., 2023). Occludin, the first integral membrane protein discovered in tight junction fibers, influences the permeability of the intestinal epithelial cell barrier, modulating the entry of macromolecules (McCarthy et al., 1996; Mazzon et al., 2002). FABP2 expression is inversely correlated with intestinal functional integrity, and its release is regulated by an imbalance in intestinal microbiota (Guerrant et al., 2016; Dutta et al., 2019; Stevens et al., 2018). The Zonula Occludens (ZO) protein family, comprising ZO-1, ZO-2 and ZO-3, acts as the scaffold protein of TJ and belongs to the membrane-associated guanosine kinase-like protein (Maguk) family (González-Mariscal et al., 2000). The double knockout of genes and proteins of ZO-1 and ZO-2 entirely prevents the formation of TJ chains, eliminating the selective permeability of the epithelial barrier (Umeda et al., 2006). Zonulin, the precursor protein of Haptoglobin-2 (pre-Haptoglobin-2), serves as the sole known regulator and marker of intestinal tight junctions. Its upregulation often signifies the disruption of tight junction structures and increased intestinal permeability (Fasano et al., 2000; Clemente et al., 2003; Tripathi et al., 2009; Fasano, 2012). Research indicates that exposure of the small intestine to Gram-negative bacteria induces the secretion of Zonulin, resulting in decreased transmembrane resistance of the mammalian small intestine and enterocyte monolayer. This downregulates the mRNA expression of intestinal tight junction-related proteins, leading to intestinal permeability increased and impaired intestinal barrier function (El Asmar et al., 2002; He et al., 2022). When the intestinal cell barrier is compromised, it becomes more susceptible to invasion by pathogenic microorganisms, fostering the colonization of pathogenic bacteria and triggering inflammation. This vulnerability is a direct contributor to the diarrhea associated with yak colibacillosis.

*Lactobacillus* (LAB) stands as a prevalent intestinal probiotic in mammals. exerting its influence through the regulation of host intestinal ecology and the intricate interplay among microbial populations (Huang et al., 2022). Existing research underscores the capacity of probiotics, including LAB, to mitigate intestinal inflammatory damage, uphold the integrity of tight junction proteins, diminish intestinal permeability, and fortify overall intestinal barrier function (Embleton and Berrington, 2013; Liu et al., 2013; Orlando et al., 2014). LAB has demonstrated efficacy in attenuating cell apoptosis and modulating the transcription of immune response genes induced by lipopolysaccharide (LPS). Additionally, it down-regulate the expression of genes associated with inflammatory responses (Vale et al., 2023). Notably, LAB strains isolated from newborn calf feces exhibit the potential for co-agglutinate with *E. coli*, thereby conferring resistance against intestinal damage caused by the latter. (Chouraddi et al., 2023). Moreover, probiotic interventions, characterized by a natural symbiosis between probiotics and their host, circumvent concerns related to drug resistance, drug residues, and aberrant host immunity. As such, this approach emerges as a secure and environmentally sustainable means of addressing colibacillosis in yaks (Bernard, 2023; Zaib et al., 2023).

Despite the wealth of literature documenting the preventive and therapeutic efficacy of LAB in intestinal diseases, limited attention has been directed toward its application in preventing and treating colibacillosis in yaks. Consequently, the present study employs an *in vitro* culture model utilizing yak epithelial cells to investigate LAB’s protective effects on the intestinal tract, particularly its resistance to *E. coli* O78. The study seeks to ascertain whether the observed effects are intricately linked to the regulation of the Zonulin protein. By doing so, it aspires to furnish both theoretical and technical underpinnings for the application of LAB in the treatment of yak colibacillosis.

## 2 Materials and methods

### 2.1 Preparation of pathogenic *E. coli* and LAB

Pathogenic *E. coli* O78 was sourced from diarrhea-afflicted yaks in Linzhi City, Xizang Autonomous Region, and preserved by the Clinical key Laboratory of the School of Animal Science, Tibet Agricultural and Animal Husbandry University. The bacteria strains were reanimated, inoculated on nutrient Agar, and cultured at 37°C for 24 hours. Single colonies were then transferred to nutritious broth and cultured overnight in a shaker incubator at 37°C. Strain detection employed Eosin-Methylene Blue Agar, and the colony-forming unit (CFU) counting method determined the required strain concentration.

Lactobacillus yoelii Lac-2, isolated from yaks, was also preserved by the Clinical key Laboratory of the School of Animal Science, Tibet Agricultural and Animal Husbandry University. *In vitro* bacteriostatic tests demonstrated the strain’s efficacy against *E. coli*, Salmonella, and Staphylococci (Wu et al., 2018). Lactobacillus was reanimated, inoculated on MRS Agar medium, and a single colony was selected and inoculated into MRS liquid medium at 37°C for 24 hours. Strain concentration was determined using the CFU counting method.

### 2.2 Cell culture and grouping

Yak intestinal epithelial cells were isolated from 30-60-day-old yak fetuses obtained from Linzhi slaughterhouse. Intestinal tissues were dissected into 1mm^3^ and washed with aseptic PBS. The yak intestinal epithelial cells were cultured with DMEM-F12 (Invitrogen, CA, USA) supplemented with 10%FBS (PAN, Adenbach, Germany) in 5%CO_2_ and 37°C incubators.

ABC staining kit (SA1002, Boster, Wuhan, China) was employed to detect the binding of Anti-Cytokeratin 18 antibody (BB12213553, Bioss, Beijing, China). Post-digestion, the cells were cultured on cell slides in a six-well plate. The cells were fixed with pre-cooled acetone at 4°C for 10 minutes, washed thrice with PBS, treated with 3% H2O2 deionized water for 10 minutes, again washed thrice with PBS, and then incubated with 5% BSA blocking solution dropwise at 37°C for 30 minutes followed by spin drying. CK18 keratin antibody was added overnight at 4°C, rinsed thrice with PBS, followed by the addition of biotin-labeled goat antibody IgG and incubation at 37°C for 30 minutes. After rinsing thrice with PBS, SABC was added and incubated at 37°C for 30 minutes, followed by thrice rinsing with PBS. The chromogenic solution DAB was added, and color development was monitored under a microscope (AE31E Trinocular 100W, Motic, China). After completion of color development, slides were rinsed with tap water and sealed with neutral gum.

Post-trypsin digestion, the cells were transferred to a 0.4 μm pore diameter PC membrane Transwell chamber for culture, with a cell count of 1 × 10^5^ cells per well. After 2 days, the medium was replaced, and cells were cultured for an additional 12 hours. The groups were divided into four: Control group (Control, normal culture), Model group (Model,1 × 10^5^CFU *E. coli* for 4 hours), Low Dose Lactobacillus group (LLAB, 1 × 10^5^CFU *E. coli* and 1 × 10^5^CFU LAB for 4 hours), High Dose Lactobacillus group (HLAB,1 × 10^5^CFU *E. coli* and 1 × 10^7^CFU LAB for 4 hours).

### 2.3 Detection of epithelial cell barrier permeability

The integrity of epithelial cell barrier was assessed using transmembrane resistance (TEER), FITC-D4 flux and bacterial translocation. Transmembrane resistance was measured with an electrical resistance system (Millipore, MA, USA). After adding FITC-D4 for 2 hours to the upper chamber of each group, the fluorescence value of the culture medium in the lower chamber was measured. After 4 hours, the lower chamber culture medium was collected for colony-forming unit calculation (CFU) to quantify bacterial translocation.

### 2.4 Measurement of mRNA expression of MUCIN and TJ proteins

The mRNA expression levels of MUC1, MUC2, Zonulin, FABP2, Occludin, ZO-1 were determined using quantitative reverse transcription polymerase chain reaction (RT-qPCR). Total RNA was extracted using the RNA-easy Isolation Reagent kit (R701, Vazyme, Nanjing, China), and its purity, integrity and concentration were measured by NanoDrop (Thermo Fisher Scientific, Wilmington, NC, USA). Complementary DNA synthesis was performed from 1 μg of total RNA using a first-strand cDNA synthesis kit (K1622, Thermo Fisher Scientific, Wilmington, NC, USA) following the kit protocol. Primers were synthesized by Tsingke Biotech (Tsingke, Beijing, China), and the primer sequences are shown in **Table 1**. Fluorescence (SYBR Green Master Mix, A25742, Thermo Fisher Scientific, Wilmington, NC, USA) was measured using ABI Prism 7500 Real-Time PCR System (Applied Biosystems, CA, USA). The cycle conditions are 95°C for 3 min and 40 cycles of 95°C for 10 s, and 60°C for 30 s. Using GAPDH as the internal reference, the relative expression of gene mRNA was calculated using the 2^-ΔΔCt^ method.

**Table 1:** Real-time PCR Primer.

Primer name	Primer sequence	Product length
MUC1-F	TTGCGCTGGCCATCATCTAT	
MUC1-R	AAGTGGCTGCCAGGTTTGTA	237
MUC2-F	AAGCAGACCTGCCTGAAGAC	
MUC2-R	CAGGTTCACCGTCTGCTCAT	108
FABP2-F	AGACCATGGCGTTTGATGGT	
FABP2-R	TTCAGTTCCATCTGCGAGGC	239
Occludin-F	GTTTGGTTCGCTGGGAGAAGA	
Occludin-R	CATTGGTCGAACGTGCATCTC	200
zonulin-F	CCAAGTACCAGGACGACACC	
zonulin-R	ACCATACTCAGCCACAGCAC	131
ZO1-F	GACCATCGTCTGCGCTATGA	
ZO1-R	CTCTGGTGAGCACGGATTGT	565
GAPDH-F	ACAGTCAAGGCAGAGAACGG	
GAPDH-R	CTCGTGGTTCACACCCATCA	240

### 2.5 Measurement of protein expression of MUCIN and TJ proteins

Cells in each group were washed with PBS, lysed with RIPA Lysis Buffer (BL504A, Labgic, Beijing, China) on ice for 30 minutes, and centrifuge at 4°C, 12000 rpm for 10 minutes to collect the supernatant. The protein concentration and quality are determined by NanoDrop, then 5 × Loading Buffer was added to prepare the protein lysate. The total protein of each group was separated by 10% SDS-PAGE, the protein was transferred to NC membrane using the wet transfer method, and sealed with 5% skim milk on the shaker for 1 h. After the first antibody (Zonulin, A1571, Abclonal, China; Occludin, A12621, Abclonal, China; MUC1, A21726, Abclonal, China; MUC2, A14659, Abclonal, China; FABP2, A1621, Abclonal, China; ZO-1, A0659, Abclonal, China; β-Actin, AC026, Abclonal, China) was incubated for 2 hours, the membrane was washed with 1 × TBST for 5 times and then washed again for 5 times with 1 × TBST after the second antibody (HRP Goat Anti Rabbit IgG H+L, AS014, Abclonal, China) was incubated for 1 h. The gray value of the bands was obtained by the chemiluminescence method, and β-actin was used as the internal reference. The gray value of the bands was analyzed by ImageJ software for standardized processing.

### 2.6 Statistical analysis of data

The experiment was repeated more than 3 times in each group, and the data was expressed as mean ± standard deviation. GraphPadPrism9.0 was used to analyze the one-way ANOVA of all data, and then Bonferroni test was used to compare the mean of each group. *P*<0.05, it is considered that the difference is statistically significant.

## 3 Results

### 3.1 Epithelial cell identification

**Figure 1:**
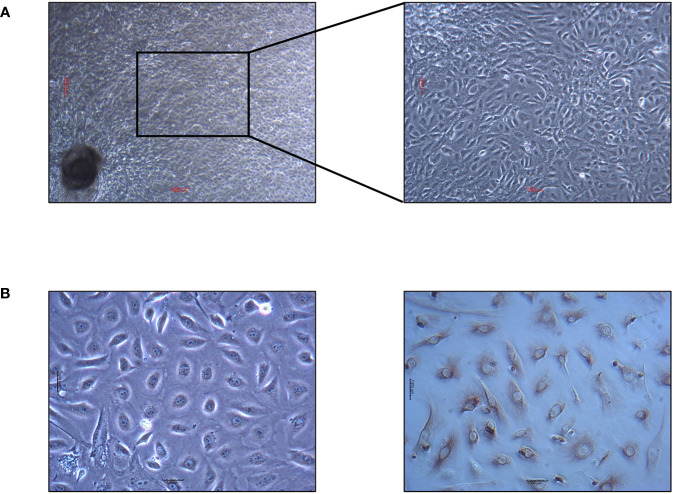
Yak intestinal epithelial cells cultured by tissue block culture method and identification results of keratin staining. **(A)** Primary yak small intestinal epithelial cells. The scale in the figure is 5000μm. **(B)** Identification results of keratin CK18 by ABC staining. The scale in the figure is 1000μm.

Yak intestinal epithelial cells isolated using the tissue block culture method are depicted in **Figure 1**. staining for CK18 with the ABC kit revealed nuclei of the epithelial cells unstained, while the surrounding nuclei were stained brown, confirming the cultured cells from yak small intestine tissue blocks as epithelial cells.

### 3.2 Detection of epithelial cell barrier function

**Figure 2:**
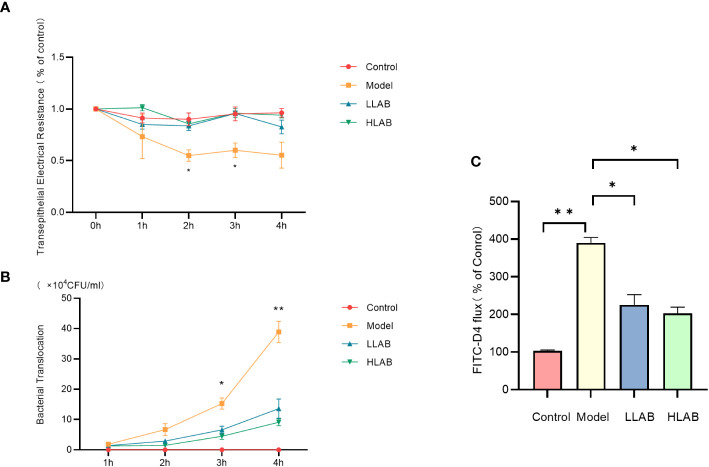
Effect of LAB on barrier function of epithelial cells infected with *E coli* O78. **(A)** Transmembrane resistance (TEER) statistical analysis. **(B)** Bacterial translocation statistical analysis. **(C)** FD4 through statistical analysis. * *P*<0.05, ** *P*<0.01.

The transmembrane resistance of Model group began to decrease at 1 h, reaching about 50% of the control group. The difference was significant at 2 h and 3 h (*P*<0.05). The transmembrane resistance of LLAB and HLAB groups remained at normal levels at all times, showing no significant difference compared with control group(*P*>0.05) (**Figure 2A**). Throughout the duration, the bacterial translocation in the Model group was over twice that of the LLAB and HLAB groups, increasing with time. LAB addition reduced bacterial translocation, with HLAB showing slightly lower levels than LLAB (**Figure 2B**). At 4 h, the FD4 permeability of Model group was significantly higher than that of Control group (*P*<0.05), while the LLAB and HLAB groups was exhibited significantly lower permeability than the Model group(*P*<0.05) (**Figure 2C**). Overall, indicators of intestinal cell barrier permeability suggest that LAB addition has a positive therapeutic effect on barrier function.

### 3.3 mRNA expression of mucin and tight junction protein

**Figure 3:**
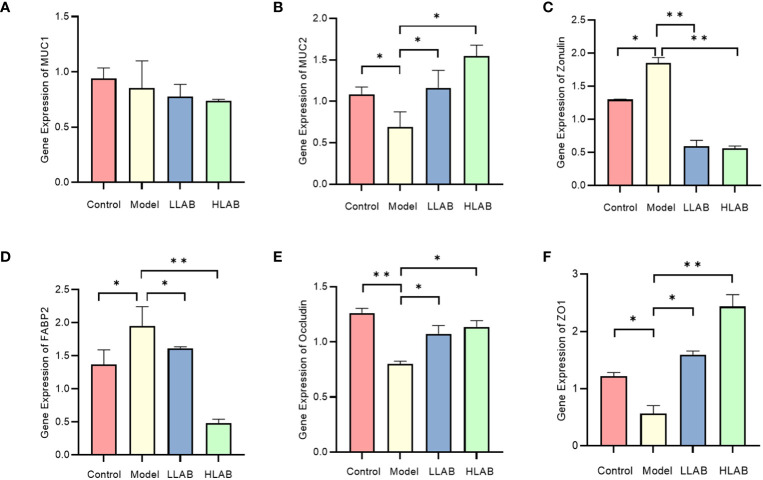
The relative expression level of mucin and tight junction protein mRNA. **(A–F)** Statistical analysis of relative expression level of MUC1, MUC2, Zonulin, FABP2, Occludin, ZO-1 mRNA. * *P*<0.05, ** *P*<0.01.

After RT-qPCR of mucin and tight junction protein in each group, MUC1 exhibited no significant change. The mRNA levels of MUC2 and ZO-1 in Model group decreased significantly, with Occludin mRNA levels showing an significant decrease. Zonulin and FABP2 mRNA levels increased significantly. LAB addition demonstrated a therapeutic effect on the abnormal expression of these genes caused by *E. coli*, except MUC1. Both low-dose and high-dose LAB significantly decreased the mRNA expression level of Zonulin in Model group. Compared with Model group, the expression levels of FABP2 and ZO-1 mRNA in HLAB group were significantly decreased (*P*<0.0l) (**Figure 3**).

### 3.4 Expression of mucin and tight junction protein

**Figure 4:**
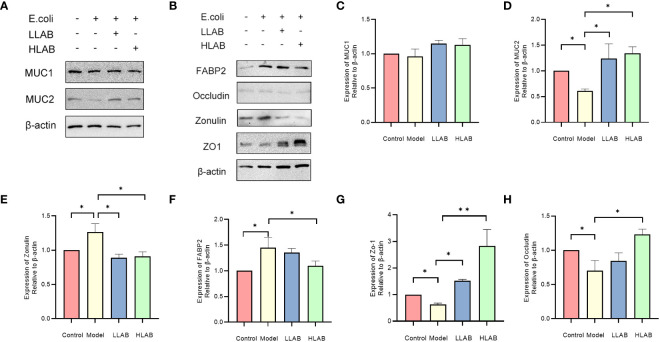
Statistical analysis of the expression of mucin and tight junction associated protein in intestinal epithelial cells. **(A)** Chemiluminescence gray image of mucin MUC1 and MCU2. **(B)** Chemiluminescence gray image of FABP2, Occludin, Zonulin, ZO-1 proteins. **(C–H)** Statistical analysis of protein expression levels of MUC1, MUC2, Zonulin, FABP2 and ZO-1,Occludin. * *P*<0.05, ** *P*<0.01.

Results of Western blotting for MUCIN and TJ proteins are shown in **Figure 4**. Consistent with mRNA expression, there was no difference in MUC1 protein among the groups (*P*>0.05) (**Figure 4C**). In Model group, the addition of *E. coli* significantly decreased the expression of MUC2, ZO-1 and Occludin protein(*P*<0.05), and up-regulated the expression of Zonulin and FABP2 protein(*P*<0.05) (**Figures 4E, F**), aligning with RT-qPCR results. In LLAB and HLAB groups, the abnormal expression of these proteins was restored by LAB addition, with ZO-1 and Occludin proteins exhibiting LAB dose dependence. LAB protected TJ protein and skeleton (**Figures 4G, H**). For MUC2, the addition of LAB enhanced the stability of the mucous layer of intestinal cells, rendering it ineffective for *E. coli* to disrupt the mucous layer (**Figure 4D**).

## 4 Discussion

In this experiment, we employed *in vitro* cultured yak intestinal cells to simulate the impact of pathogenic *E. coli* infection on intestinal barrier function and studied the therapeutic effect of adding LAB to the intestinal barrier. The decrease in TEER caused by *E. coli* infection indicates insufficient intestinal epithelium to maintain normal barrier function, with subsequent damage to the tight junction structure between epithelial cells, as confirmed by subsequent TJ protein detection. The increase in FD4 permeability signifies an increase in intestinal paracellular transport volume. Correspondingly, bacterial translocation increases, causing the loss of selective permeability in epithelial cells (González-Quilen et al., 2021). The addition of LAB restored normal transmembrane resistance, weakened FD4 flux, and reduced the ability of pathogenic bacteria to breach the cellular barrier. This aligns with previous studies, demonstrating LAB’s positive impact on barrier function (González-Orozco et al., 2023; Song et al., 2023). To elucidate the mechanism of LAB, we further explored the relationship between LAB and TJ structure and the intestinal mucus layer.

The intestinal tract comprises a physical barrier composed of the TJ structure and a chemical barrier formed by the mucus layer. The former is regulated by TJ proteins, and the latter by MUCIN proteins (Halpern and Denning, 2015). Pathogenic bacteria infection down-regulated ZO-1 and Occludin expression. ZO-1, located at the top of epithelial cells, is a crucial constituent protein of the tight junction structure (Pizzuti et al., 2004; Strauss et al., 2021). We speculate that pathogenic *E. coli* led to ZO-1 protein destruction, resulting in cytoskeleton rearrangement and tight junction structure decomposition. This is generally associated with ZO-1 and myosin 1C phosphorylation (Sturgeon et al., 2017; Pederzolli et al., 2018; Zeng et al., 2023). Similarly, Occludin detection results showed a decrease in Occludin expression caused by pathogenic *E. coli*, leading to the translocation of Occludin in the TJ structure and increased intestinal barrier permeability (Mazzon et al., 2002). After LAB treatment, the expression of ZO-1 and Occludin proteins returned to normal, indicating that LAB can help intestinal epithelial cells resist the destruction of tight junction structure caused by *E. coli*. We also observed changes in FABP2 levels in different treatment groups. The up-regulation of FABP2 expression, negatively correlated with intestinal functional integrity, occurred when *E. coli* infected epithelial cells (Guerrant et al., 2016; Dutta et al., 2019; Bruce et al., 2018). After LAB addition, FABP2 expression returned to control group levels, suggesting that LAB positively affects abnormal FABP2 expression caused by *E. coli* infection.

Zonulin, considered a tight junction regulator, is associated with conditions such as diabetes and bipolar disorder (Craig Sturgeon and Fasano, 2016; Wood Heickman et al., 2020). We detected Zonulin expression in different treatment groups, noting that *E. coli* treatment resulted in Zonulin upregulation, generally considered a sign of intestinal tight junction destruction (Asbjornsdottir et al., 2023; Zhao et al., 2023). This aligns with the down-regulation of tight junction-related proteins ZO-1 and Occludin observed previously. Studies have shown that Zonulin up-regulation-induced changes in tight junction permeability are part of autologous maintenance of intestinal homeostasis, including specific immune response regulation and increased intestinal permeability (Fasano and Shea-Donohue, 2005; Sturgeon and Fasano, 2016; Sturgeon et al., 2017). Zonulin and tight junction protein levels returned to normal in the LAB treatment group, suggesting that LAB may reduce Zonulin’s down-regulation on related tight junction proteins and protect the intestinal barrier by affecting the up-regulation of intestinal Zonulin expression caused by Gram-negative bacteria (Serek and Oleksy-Wawrzyniak, 2021). Further research is needed to determine whether LAB directly affects Zonulin expression to regulate tight junction protein expression.

Mucin MUC1 and MUC2 present in the intestinal mucous layer can adhere to pathogenic bacteria, protecting the intestinal epithelial mucosa (Cox et al., 2023). Our results showed that after *E. coli* infection, MUC2 protein was significantly down-regulated, weakening intestinal adhesion and pathogen resistance. However, its function recovered significantly after Lactobacillus treatment, proving that Lactobacillus restored mucous layer function, increased mucin expression, and Lactobacillus extracellular polysaccharides (LAB-EPS) exhibited anti-inflammatory and antibacterial activities. This inhibits *E. coli* growth in the intestinal tract, improving intestinal flora balance and immune function (Wang et al., 2023; Yang et al., 2023a; Yang et al., 2023b). However, there is no difference in the expression of MUC1 among different treatment groups, necessitating further studies to determine whether *E. coli* no effect on intestinal MUC1.

In summary, Lactobacillus can protect intestinal barrier function, improving the down-regulation of tight junction proteins caused by pathogens, balancing the intestinal flora structure, maintaining normal intestinal permeability. Its function may stem from Lactobacillus’s regulation of Zonulin protein, affecting the overall TJ structure. This study provides new theoretical support for probiotic treatment of yak colibacillosis, potentially contributing to the economic benefits of the yak breeding industry. Future research will explore whether LAB can still regulate TJ protein after Zonulin gene knockout.

## 5 Conclusion

LAB can help intestinal epithelial cells resist the destruction of the intestinal barrier caused by *E. coli*. This therapeutic mechanism involves the regulation of MUC2 protein by LAB, enhancing the adhesion function of the intestinal mucous layer. Additionally, LAB regulates TJ-related proteins, enhancing the stability of the TJ between intestinal epithelial cells. The function may also be attributed to the regulation of Zonulin protein by LAB, affecting the overall TJ structure. This study offers new theoretical support for probiotic treatment of yak colibacillosis, with potential contributions to the economic benefits of the yak breeding industry. Future investigations will further verify LAB’s regulatory role in TJ proteins after Zonulin gene knockout.

## Data availability statement

The original contributions presented in the study are included in the article/supplementary materials, further inquiries can be directed to the corresponding author/s.

## Ethics statement

The requirement of ethical approval was waived by Tibet Agriculture and Animal Husbandry University for the studies involving animals because of sampling of slaughtered yaks from the slaughterhouse of Linzhi City. The studies were conducted in accordance with the local legislation and institutional requirements.

## Author contributions

JZ: Data curation, Methodology, Writing – original draft, Writing – review & editing. XR: Methodology, Writing – review & editing. SW: Methodology, Writing – review & editing. RL: Methodology, Writing – review & editing. BS: Methodology, Writing – review & editing. HD: Writing – review & editing, Methodology. QW: Conceptualization, Supervision, Writing – review & editing.
